# Autophagy: Mechanisms and therapeutic perspectives in intestinal metaplasia (Review)

**DOI:** 10.3892/mmr.2026.13999

**Published:** 2026-08-21

**Authors:** Laifeng Wu, Lingchen Liao, Ziwei Xia, Qiao Zeng, An Huang, Zhiyong Cao

**Affiliations:** 1 Zhuang Medical College, Guangxi University of Chinese Medicine, Nanning, Guangxi Zhuang Autonomous Region 530001, P.R. China; 2 Institute of Traditional Chinese and Zhuang-Yao Ethnic Medicine, Guangxi University of Chinese Medicine, Nanning, Guangxi Zhuang Autonomous Region 530200, P.R. China

**Keywords:** autophagy, intestinal metaplasia, gastric precancerous lesion, mechanism of action, potential therapeutic

## Abstract

Intestinal metaplasia (IM) represents a key stage in the progression of gastric mucosal lesions to gastric cancer, marked by a complex molecular pathogenesis and a lack of effective interventional strategies. Autophagy, a key mechanism for maintaining cell homeostasis, is closely associated with the onset and progression of IM. The present review aimed to summarize the role of autophagy in IM by integrating its association with risk factors and pathogenic mechanisms. The present study evaluated the impact of autophagy on inflammation, immune responses and cellular fate determination during IM. The present study summarized potential autophagy-mediated therapeutic agents for IM treatment and future research directions and existing limitations, aiming to provide novel insight for preventing and managing this premalignant condition.

## 1. Introduction

Gastric cancer (GC) is one of the most common malignant neoplasms affecting the digestive tract worldwide. In 2020, the Global Cancer Statistics (GLOBOCAN) reported ~1.089 million new cases and 768,000 deaths globally, with its incidence and mortality rates ranking the fifth and fourth, respectively, among all malignancy, thus representing a notable public health threat (1). The widely accepted Correa cascade hypothesis suggests that GC develops through a multi-stage process driven by chronic inflammation, advancing sequentially from gastric mucosal atrophy to intestinal metaplasia (IM), dysplasia and culminating in invasive adenocarcinoma (2). In this progression, IM is a crucial phase in the inflammation-cancer transformation, with the complete subtype regarded as an irreversible threshold for malignant transformation (3). Therefore, preventing the onset and progression of IM is strategically key for the early prevention of GC. The pathological hallmark of IM is the substitution of acid-secreting cells or antral mucosa with intestinal mucosal epithelium, which comprises Paneth, goblet and absorptive cells (4). Although the exact molecular mechanisms for this process are unclear, autophagy may serve a dual role in IM development by mediating risk factors and pathogenic mechanisms (5,6).

Autophagy is a highly conserved degradation and recycling system in eukaryotic cells. This key process involves the sequestration of impaired organelles, misfolded proteins and pathogens within double-membrane vesicles called autophagosomes, which are transported to lysosomes for degradation, thereby maintaining cell homeostasis (7). Under conditions such as nutrient deprivation, infection or oxidative stress, autophagy inhibits the accumulation of deleterious substances and maintains energy supply by clearing damaged components and degrading macromolecules to release metabolic substrates, including amino acids (8,9). Prolonged stress or dysregulation of autophagic function cause autophagy to transition from a protective mechanism to a pathological contributor, leading to the excessive degradation of key organelles and functional proteins. This disruption compromises cell structural and functional integrity and may provide energy to compromised or malignant cells, thereby facilitating their survival and proliferation (10,11).

Autophagy is classified into three types based on the mechanism of substrate delivery to lysosomes: Macroautophagy, microautophagy and chaperone-mediated autophagy. Macroautophagy (hereafter referred to as autophagy) is the most extensively studied form (12,13). The molecular process of autophagy comprises five stages: Initiation, nucleation, elongation, fusion and degradation (14). This process involves core regulators, including autophagy-related protein (ATG) protein family, Unc-51-like autophagy-activating kinase 1 kinase and PI3K-III/Beclin-1 complexes and ATG5-ATG12-ATG16L1 ubiquitination system, and is regulated by key signaling pathways, including PI3K and mTOR (15,16).

Dysregulated autophagy is extensively implicated in pathological processes, including neurodegenerative disease, infections, immune disorder and malignant tumors (17,18). Although research has partially clarified the roles of autophagy in GC pathogenesis and progression (19), its mechanisms of action in gastric precancerous lesions, especially during the stage of IM, remain insufficiently summarized and analyzed. The present review aims to address the initiation mechanisms of autophagy driven by risk factors associated with IM, the potential roles of autophagy during IM progression and autophagy-mediated therapeutic strategies for IM. By systematically analyzing research advancements, this article aims to provide a novel theoretical foundation and strategic insights for clarifying the molecular mechanisms of IM and investigating preventive interventions for precancerous gastric lesions.

## 2. Autophagy mediates promotion of IM by associated risk factors

The onset of IM is associated with risk factors, including *Helicobacter pylori* infection, aging, chronic alcohol consumption, smoking, poor diet and bile reflux (20–22). Continuous stimulation of the gastric mucosa by these factors prompts autophagy to serve as a vital cytoprotective mechanism, initially preserving gastric mucosal homeostasis by eliminating damaged cell components. However, prolonged exposure to these pathogenic factors may lead to autophagy dysfunction, thereby facilitating IM initiation and progression (23–25). In addition, multiple studies have shown that autophagy, through the regulation of key targets, including vacuolating cytotoxin A, p62, ATG2B, ATG5 and ATG12, and their related signaling pathways, mediates the promotion of intestinal metaplasia through risk factors such as *H. pylori*, N-Methyl-N'-nitro-N-nitrosoguanidine, high-fat diet and deoxycholic acid [Table I (26–31)].

## 3. Potential role of autophagy in IM

The regulation of autophagic activity is associated with the onset and progression of diseases, including cancer and neurodegenerative disorders (32). Research has demonstrated notable dysregulation of autophagy in the IM stage, which involves inflammatory responses (33), immune reactions (34) and changes in cell types (35). A comprehensive investigation of the regulatory roles of autophagy is key for the early intervention of IM.

### Autophagy and inflammatory response

**Figure 1.**
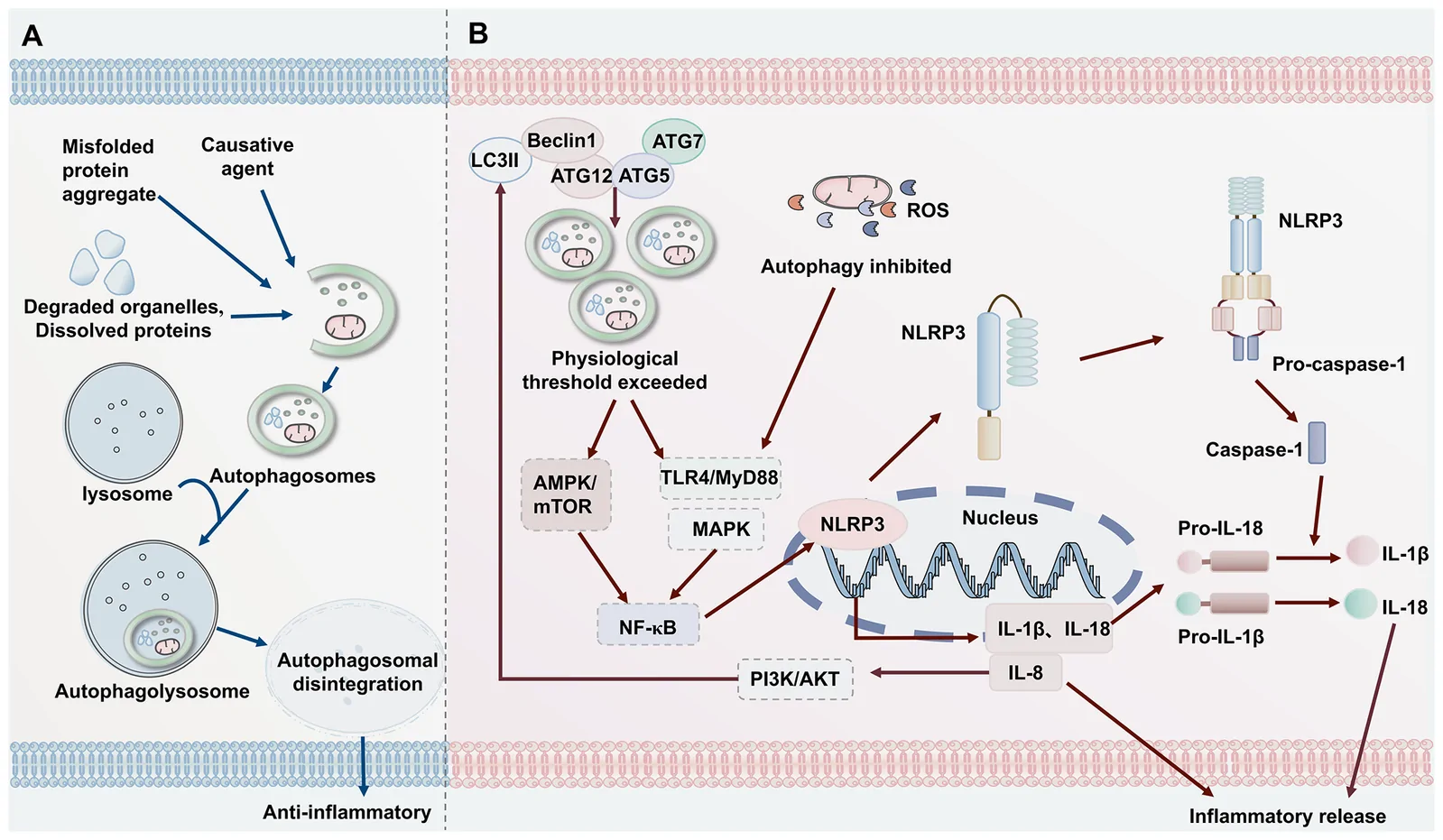
Dual role of autophagy in the initiation and resolution of inflammation. (A) In the early stages of disease, autophagy serves as a cell-autonomous anti-inflammatory mechanism by encapsulating and degrading intracellular pathogens, misfolded proteins and other endogenous inflammatory signals within autophagosomes. These cargoes are cleared via the autophagosome-lysosomal pathway, thereby suppressing the activation of pro-inflammatory signaling cascades at their source. (B) Under sustained inflammatory stimulation, inhibition of autophagy amplifies signaling through the TLR4/MyD88/MAPK/NF-κB pathway and hyperactivates the NLRP3 inflammasome, promoting its complex assembly and caspase-1 activation. This leads to maturation and release of IL-1β and IL-18. Conversely, excessive autophagy triggers an energy crisis through organelle degradation, which activates NF-κB via the AMPK/mTOR pathway, further exacerbating inflammatory responses. Additionally, IL-8 enhances cell invasiveness via PI3K/AKT activation and upregulates autophagy markers such as LC3II, ATG5 and 7 and the ATG12-ATG5 conjugate, thereby promoting pathological hyperactivation of autophagy. ROS, reactive oxygen species.

Inflammation is a fundamental driver in the progression of gastric precancerous lesions, with its pathological influence extending throughout IM and the Correa cascade (2). During the initial stages of the disease, autophagy exerts cell-autonomous (an intrinsic and proactive self-protective response mounted by the epithelial cell itself upon initial exposure to inflammatory stimuli) anti-inflammatory effects by eliminating intracellular pathogens, damage-associated molecular patterns released from damaged organelles and misfolded protein aggregates, sources of inflammatory signals, thereby effectively inhibiting the activation of pro-inflammatory signaling pathways, including the NLRP3 inflammasome (Fig. 1A) (36,37). Conversely, when autophagy is impaired or structurally defective, this protective mechanism is disrupted, leading to abnormal activation and persistence of inflammatory responses. Dysfunction of the autophagy-lysosome pathway may constitute a common pathological basis for inflammatory diseases (38). The transcription factors TFEB and TFE3, as primary regulators of macroautophagy/autophagy and lysosomal function, may act as key molecular hubs linking these pathological processes (39).

Prolonged inflammatory stimulation leads to impaired autophagic flux, resulting in excessive activation of the NLRP3 inflammasome and facilitating the formation and oligomerization of inflammasome complexes (40,41). These complexes include nucleotide-binding oligomerization domain-like receptors, adaptor proteins and pro-caspase-1 (42). The activation of these inflammasome complexes causes the proteolytic cleavage of the zymogen pro-caspase-1 into its enzymatically active form and catalyzes the maturation and release of pro-inflammatory cytokines IL-1β and IL-18 (43,44). However, when autophagic activity exceeds physiological thresholds, its compensatory enhancement may lead to a pathological state of excessive autophagy. Excessive degradation of organelles may precipitate an energy metabolic crisis, activating the NF-κB signaling pathway through the AMPK/mTOR pathway and initiating a cascade of inflammatory responses (45,46). Moreover, the impaired autophagic flux enhances the activation of the TLR4/MyD88, MAPK and downstream NF-κB signaling pathways and exacerbates the release of pro-inflammatory factors (IL-6, IL-1β, tumor necrosis factor-α and IL-8) and other factors, including chemokines CCL7 and CXCL16 (47–49). Notably, IL-8 can enhance autophagy and cell invasiveness by regulating the phosphorylation of the PI3K/AKT pathway. Elevated levels of IL-8 are associated with a marked increase in the expression of autophagic markers LC3II, ATG5, ATG7, the apoptotic factor Beclin1 and the ATG12-ATG5 complex (50). Furthermore, NF-κB initiates the activation of the NLRP3 inflammasome by upregulating pro-IL-1β and NLRP3 expression, thereby establishing a pro-carcinogenic feedback loop (Fig. 1B) (51).

### Autophagy and immune regulation

**Figure 2.**
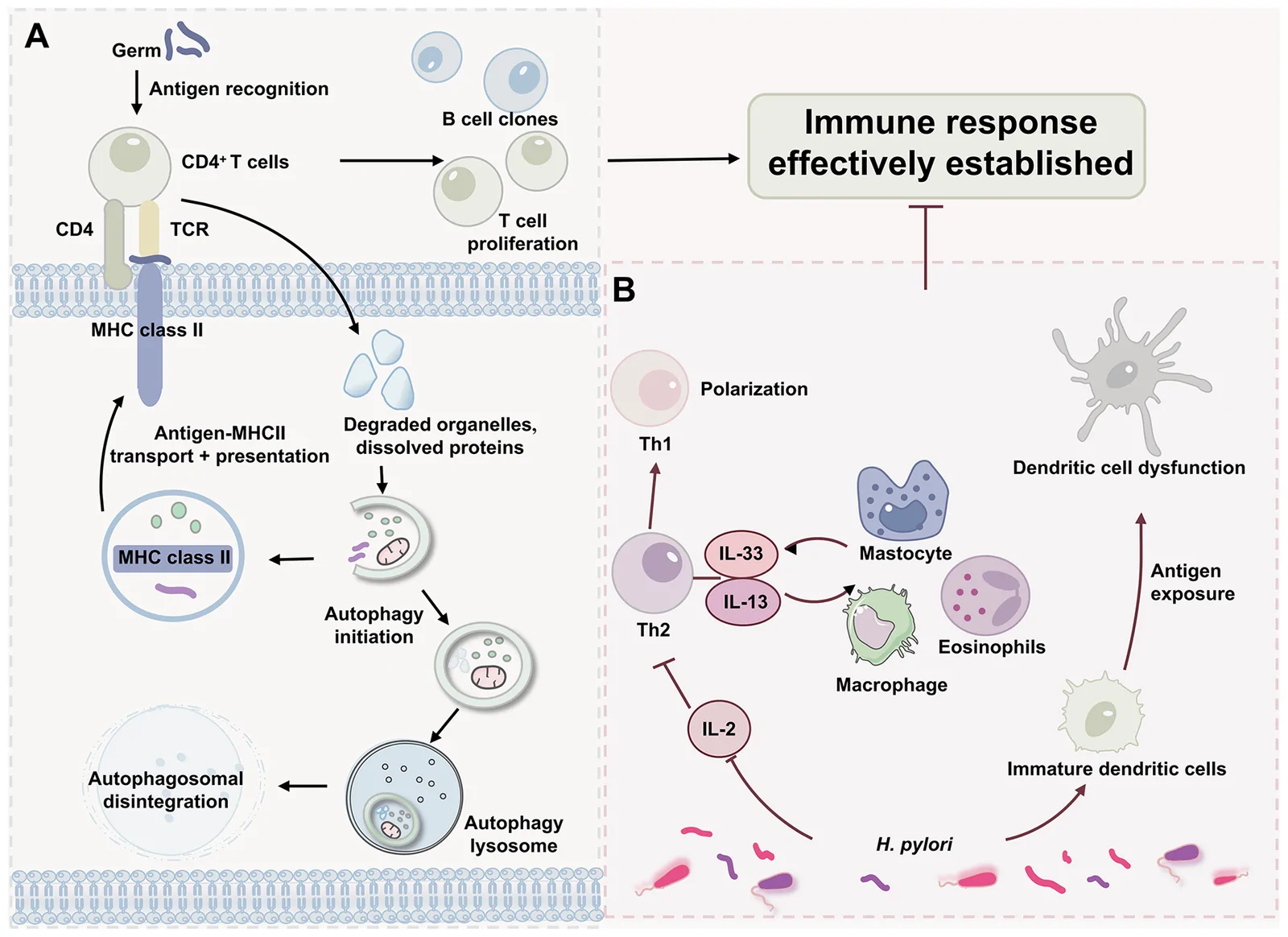
Mechanisms of autophagy in immune regulation and *H. pylori* response. (A) Under physiological conditions, autophagy contributes to immune homeostasis by recognizing and degrading bacterial pathogens and facilitating antigen presentation via MHC class II molecules. This enhances CD4⁺T cell responses against pathogens and tumors, promotes self-tolerance and supports clonal expansion of T and B cells, thereby strengthening overall immune competence. (B) In the context of *H. pylori* infection, the bacterium suppresses IL-2 production and Th2 responses, skewing immunity toward Th1 polarization. Th2-associated cytokines such as IL-33 and IL-13 contribute to the progression of gastritis and IM through interactions with macrophages, mast cells and eosinophils. Although *H. pylori* potently activates and matures immature dendritic cells, chronic antigen exposure may lead to dendritic exhaustion, impairing the establishment of effective immune responses. *H. pylori, Helicobacter pylori;* MHC, major histocompatibility complex; Th, T helper; IM, intestinal metaplasia; TCR, T cell receptor.

Autophagy is a key mechanism by which cells manage endogenous and exogenous stress, thereby enhancing innate and adaptive immunity. Autophagy enhances innate pathogen detection, antigen presentation, pathogen clearance and lymphocyte expansion (52,53). In the context of innate immunity, autophagy serves as the primary defense mechanism against microbial invaders. Upon detecting invading bacterial pathogens and their associated infection signals, autophagic proteins serve as cytosolic sensors, promptly activating the autophagy pathway and initiating protective mechanisms for the host (54). When the gastric mucosa is invaded by pathogens such as *H. pylori*, the autophagy system can promptly detect the specific infection signals of bacteria, rapidly activate autophagy-associated proteins as intracellular recognition receptors and initiate the autophagy pathway to encapsulate and degrade bacteria, thereby limiting the proliferation of pathogens and mitigating damage to the gastric mucosa (55). During innate immune responses, autophagy facilitates the presentation of degradation products through the lysosomal pathway to major histocompatibility complex (MHC) class II molecules (56). This stimulates and refines the self-tolerance of the CD4^+^ T cell repertoire, enhances CD4^+^ T cell responses to pathogens and tumors and facilitates the clonal expansion of B and T cells, thereby strengthening overall immune function (Fig. 2A) (57). Conversely, autophagy is key in distinguishing self from non-self by regulating exogenous factors in antigen-presenting cells, such as MHC-antigen complexes, and endogenous factors in T cells, including cell signaling, survival, cytokine production and metabolism. This renders it a target for regulating T cell immunity (58). Furthermore, the autophagy-associated protein phosphatidylinositol 3-kinase catalytic subunit type 3/vacuolar protein sorting 34 directly affects T cell function by maintaining metabolic homeostasis (59). However, a deficiency in autophagy leads to abnormal activation of mTORC1 and c-Myc signaling pathways, which enhances glycolytic metabolism and impairs the function of regulatory T cells (Tregs) (60). Deficient Treg function may result in uncontrolled inflammatory responses. This increases the risk of peptic ulcers in *H. pylori* infection and drives the pathological remodeling of the gastric mucosa from chronic inflammation to atrophy and IM (61). The gastric mucosal microenvironment relies on autophagy-mediated antigen presentation, T cell repertoire shaping and the maintenance of Treg function (62).

Following *H. pylori* infection, these autophagy-dependent immune regulations are locally activated or dysregulated in the gastric mucosal microenvironment, influencing the establishment of protective tolerance or the progression toward chronic inflammation and carcinogenesis (62,63). This mechanism involves the modification of surface molecules and the regulation of macrophage and T cell functions to evade immune surveillance (64). *H. pylori* transforms autophagy into a pro-survival pathway by inhibiting autophagosome-lysosome clearance, which serves as a key strategy for persistent infection (65). At the immune response level, *H. pylori* components directly enhance the secretion of IFN-γ and IL-12 and simultaneously inhibit IL-2 production and the cell proliferation necessary for Th2 responses, thereby promoting Th1 polarization (61,66). This polarization state exacerbates local inflammatory damage to the gastric mucosa, induces abnormal apoptosis and repair of epithelial cells and can promote the transformation of normal gastric mucosa to IM in the long term (67). Although traditional views highlight Th1 immunity in gastritis and IM, emerging evidence indicates that the interaction network of Th2-associated cytokines (IL-33 and IL-13) with M2 macrophages, mast cells and eosinophils is an important trigger for tumorigenesis (68). This network can disrupt the integrity of the gastric mucosal barrier, thus accelerating disease progression (68). *H. pylori* can induce the strong activation and maturation of human immature dendritic cells (Fig. 2B) (69). However, continuous antigen exposure may induce functional exhaustion of dendritic cells, which affects the effective establishment of a Th1 immune response, resulting in insufficient immune clearance and persistent chronic inflammation (70). This promotes the occurrence of IM in the gastric mucosa during repeated injury and abnormal repair.

### Autophagy in cell development and differentiation

**Figure 3.**
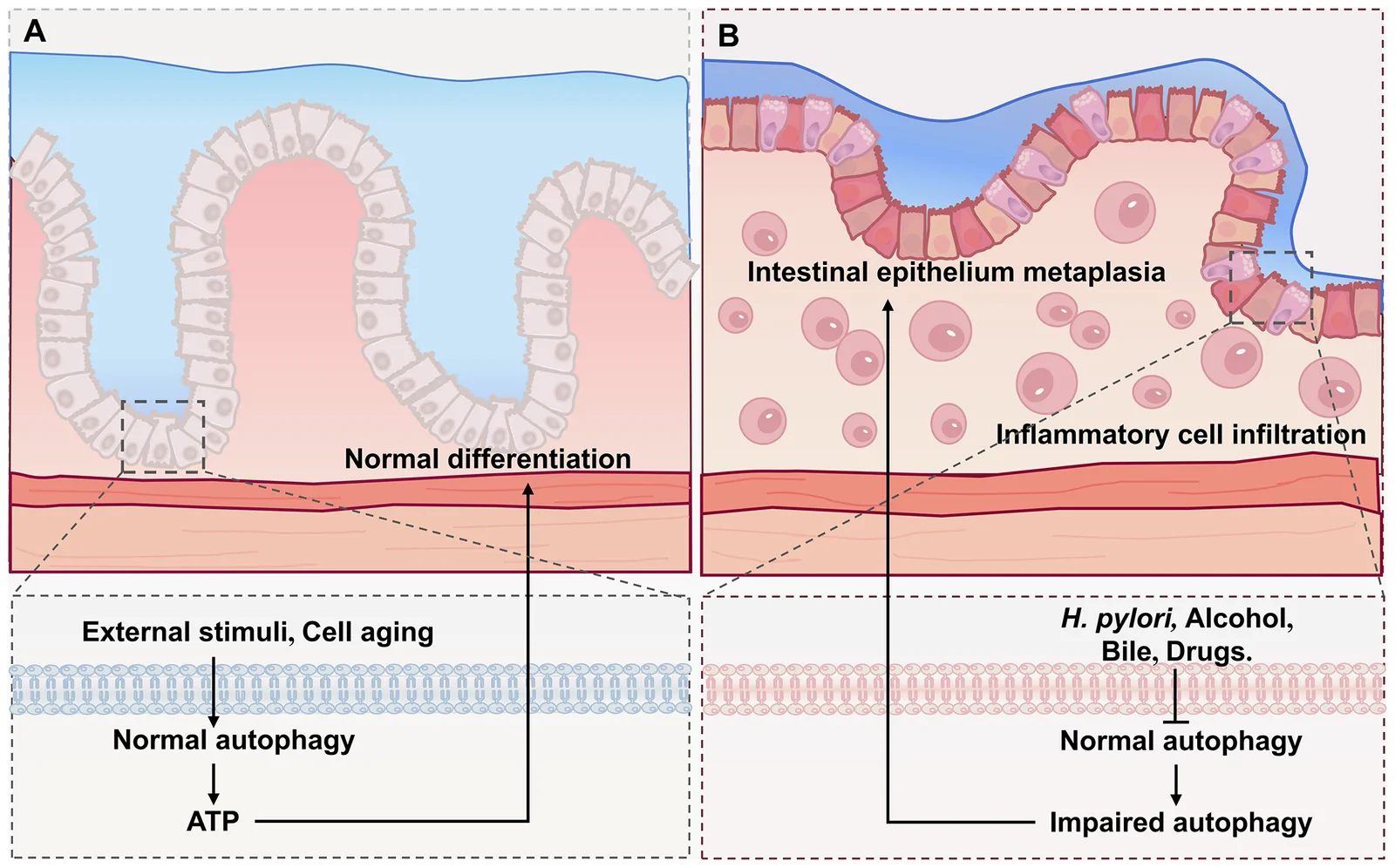
Dual role of autophagy in gastric epithelial homeostasis and IM. (A) Under physiological conditions, autophagy is activated in gastric epithelial cells in response to aging or external stimuli. Autophagy clears damaged components and recycles them into ATP and other energetic substrates, thereby supporting cell development and differentiation. (B) Under chronic insults such as *H. pylori* infection, alcohol consumption, bile reflux or drug-induced injury, autophagy becomes dysregulated. Dysfunction compromises cellular defense mechanisms and disrupts the normal differentiation trajectory of gastric stem cells. This leads to the emergence of abnormal cell phenotypes, functional disturbance and inflammatory cell infiltration, collectively promoting IM initiation and progression. IM, intestinal metaplasia; *H. pylor, Helicobacter pylori.*

Autophagy, a highly conserved intracellular degradation mechanism, is key in various biological processes, including cell survival, death, differentiation and metabolism (71). Under normal physiological conditions, the gastric mucosa is primarily composed of gastric-type epithelial cells. Similar to most normal cells, autophagy facilitates self-renewal and differentiation by degrading excessive cell proteins and organelles, thus providing metabolic precursors and energy (Fig. 3A) (72). Autophagy serves a protective function by attenuating damage from external stimuli. For example, it protects gastric mucosal epithelial cells against ethanol-induced apoptosis and mucosal damage by inhibiting the generation of ethanol-induced reactive oxygen species, preserving antioxidant enzyme integrity and reducing lipid peroxidation (73). Additionally, signaling proteins such as IFN-γ decrease epithelial cell apoptosis by inducing autophagy and simultaneously suppressing the aberrant proliferation of gastric progenitor cells. This dual action helps maintain a balance between infection defense and carcinogenesis suppression within the gastric mucosa (74).

Chronic pathological stimulation causes secretory columnar epithelial cells (which produce gastric acid and mucus) to lose their inherent functional characteristics and differentiate into goblet cells, intestinal-type absorptive cells with microvilli and Paneth cells (75–77). This cellular remodeling necessitates enhanced degradative capacity to facilitate structural transformation (72). However, autophagy dysregulation leads to dual pathological effects. Specifically, impaired or excessively enhanced autophagic flux loses its protective effect on gastric epithelial cells and accelerates their death (78); it also disrupts the degradation-remodeling balance and weakens the normal support for cell transdifferentiation. This functional imbalance provides a pathological basis for aberrant cell transformation in IM (Fig. 3B). Inflammatory factors can trigger the deconstruction of the chief cell secretory apparatus and transcriptomic reprogramming, a process dependent on autophagy and coordinated changes in gene transcription (79,80). In the gastric mucosa, autophagy dysregulation may affect stem cell differentiation pathways, resulting in aberrant cell phenotypes and functions (81,82). In the absence of autophagy, gastric mucosal stem cells demonstrate a bias toward intestinal differentiation (83). At the molecular level, crosstalk between autophagy and signaling pathways is key for stem cell differentiation, such as influencing differentiation direction via regulation of the Wnt pathway (84) and modulating cell metabolic status and differentiation capacity through interaction with the mTOR signaling pathway (85,86). Dysregulation of these pathways in the gastric mucosa may lead to abnormal stem cell differentiation, promoting IM development (87).

The protective and pathogenic effects of autophagy on gastric mucosa, based on the evidence from inflammation, immune regulation and cell differentiation, are not absolute but depend on a molecular switch influenced by multiple threshold factors (62). At basal levels of autophagic activity, autophagy inhibits the overactivation of the NLRP3 inflammasome and NF-κB pathway by clearing pathogens, damaged organelles and misfolded proteins, thereby exerting anti-inflammatory and homeostatic functions (88,89). However, prolonged suppression or compensatory overactivation of autophagy may shift its role from protective to pathogenic (62). Specifically, the key threshold factors include the bidirectional threshold effect of oxidative stress. Low-level oxidative stress is eliminated through autophagy, thereby exerting protective effects, whereas high-level stress that exceeds autophagic capacity may result in autophagy blockage or hyper-activation, leading to organelle degradation, energy metabolism crisis, NF-κB activation and promotion of epithelial cell apoptosis and transdifferentiation (90–92). Elevated IL-8 enhances autophagic activity and cellular invasiveness through PI3K/AKT pathway activation, thereby establishing a positive-feedback loop (50). Appropriate levels of cytokines, particularly IFN-γ, in the immune microenvironment induce protective autophagy, whereas continuous abnormal elevation may disrupt immune tolerance by promoting Th1 polarization and impairing Treg function (75). Collectively, these factors determine the transition of autophagy from a protective barrier to a driver of IM.

## 4. Autophagy-mediated therapy for IM

The primary clinical interventions for IM include *H. pylori* eradication, endoscopic surveillance and surgical treatment. However, these approaches have demonstrated limited efficacy in reversing existing IM lesions and remain insufficient for specific and continuous treatment (93,94). Recently, advances in the understanding of autophagy regulation mechanisms have identified pharmacological agents and bioactive compounds, including metformin (95), Xiaojianzhong decoction (96), notoginsenoside (97) and *Celastrus orbiculatus* (98), that may exert a bidirectional regulatory role in treating IM by targeting key autophagy signaling pathways and regulating the expression of autophagy-associated proteins (Table II) (95–104). Several autophagy regulators have entered the clinical evaluation stage. For example, a prospective randomized controlled trial involving 140 non-diabetic patients with IM demonstrated that the reversal rate of IM in the metformin (500 mg/day) treatment group was 48.6%, significantly higher than 31.4% in the folic acid control group, indicating that metformin can effectively reverse IM (105). However, with the exception of metformin, most autophagy-targeted drugs remain at the preclinical stage, and, to the best of our knowledge, no additional randomized controlled trials for IM indications have been conducted (Table II).

In a precancerous gastric lesion model, histopathological examinations and western blotting confirmed increased expression of Beclin-1 and LC3II in gastric mucosa tissue, indicating altered autophagic activity during IM development (96). Another GC study demonstrated that abnormal expression of these proteins and p62 is associated with pathological progression and may possess predictive value for early-stage GC (106). These findings support the potential use of combined assessment of autophagy markers, including LC3II and Beclin-1, in gastric biopsy specimens for diagnostic and prognostic evaluation, although validation through large-scale cohort studies remains necessary. Although autophagy regulators demonstrate potential in treating IM, clinical translation faces limitations. Animal models for *H. pylori*-related gastric lesions have drawbacks in mimicking human IM and associated autophagic responses. Despite *H. pylori* being a major cause of chronic gastritis and GC, existing models cannot fully reproduce the complexity of human infection and pathological progression (107). For example, the *H. pylori*-N-nitroso-N-methylurea-induced mouse model reproduces phenotypes such as IM, neutrophil infiltration and autophagy disorder, but its 24-44-week observation window is in the early stage, failing to reflect long-term pathological changes and carcinogenic mechanisms (108). Clinical specimens are predominantly obtained from the gastric antrum, whereas animal experiments focus on the gastric corpus, decreasing the comparability between model results and human pathology (109). These limitations highlight the need to establish more clinically relevant models.

Autophagy-associated proteins participate in canonical autophagic processes and autophagy-independent signaling pathways. This multi-biological property means autophagy-targeted interventions may induce dysregulation of other pathways and potential side effects (110). IM comprises different subtypes with heterogeneous autophagic activity, which affects treatment specificity and efficacy (111,112). Notably, compounds such as metformin and notoginsenoside systemically regulate autophagy rather than specifically acting on the gastric mucosa. For example, metformin-induced systemic autophagy activation may confer metabolic benefits, but may lead to metabolic imbalance in conditions such as renal insufficiency (113,114). Similarly, notoginsenoside can protect through multi-organ autophagy regulation, but long-term administration requires caution due to potential risks of autophagy homeostasis disruption (115,116). Consequently, in long-term clinical use of these drugs, individualized risk assessment and monitoring should be performed based on pharmacokinetic characteristics, renal function and concurrent medications to avoid systemic toxicity in non-target organs.

## 5. Conclusion

The present study highlighted the dual and dynamic role of autophagy in IM initiation and progression. Autophagy provides protective effects by modulating inflammatory responses, maintaining immune homeostasis and remodeling cellular differentiation. However, autophagy dysfunction may also serve as a key driver of sustained IM progression. Consequently, autophagy represents a key entry point for understanding the mechanisms underlying IM and a potential target for intervention.

It is essential to identify key regulatory nodes of autophagy during IM progression and develop selective autophagy modulators. The specific application of induction or inhibition strategies should be examined based on the dynamic alterations in autophagic activity at various stages of the disease. Clinical studies are key to validate the efficacy and safety of autophagy-targeted interventions and determine the optimal timing and therapeutic window for these interventions. Comprehensive investigations are essential to clarify the molecular profiles and subtype-specific roles of autophagy across IM subtypes. This includes clarifying its effects independent of autophagy and understanding the sensitivities of these subtypes to autophagy modulators. Employing advanced technologies such as single-cell multi-omics and spatial transcriptomics may reveal the heterogeneity in autophagy regulation throughout pathological stages of IM, aiding in the development of subtype-specific interventional strategies.

Furthermore, IM should be considered a distinct research focus, rather than a component of gastric precancerous lesions, to enhance the understanding of its unique molecular mechanisms. It is essential to confirm autophagy modulation strategies in clinically relevant models and examine their potential to reverse IM, overcome *H. pylori* drug resistance and facilitate personalized prevention and treatment. These efforts are key for overcoming the challenges in translating basic research into clinical applications and advancing clinical implementation.

## Figures and Tables

**Table I.** Autophagy mediates the enhancement of intestinal metaplasia through associated risk factors.

A, *H. pylori* infection			

Mechanism	Targeted molecule(s)	First author, year	(Refs.)
Maturation of autolysosomes is inhibited, resulting in a general decrease in autophagic activity and the exacerbation of gastric precancerous lesions	Vacuolating cytotoxin A	Raju *et al*, 2012	(26)
Autophagy suppression leads to abnormal accumulation of p62, which facilitates ubiquitination-mediated degradation of Rad51, thereby impairing DNA repair and compromising genomic stability	p62	Xie *et al*, 2020	(27)
Upregulation of miR-30d is induced, which directly targets core autophagy genes, such as ATG2B, ATG5, ATG12, BECN1 and BNIP3L, to suppress autophagy and enhance the intracellular survival of *H. pylori* in AGS cells	ATG2B, ATG5, ATG12, BECN1, BNIP3L	Yang *et al*, 2016	(28)

**B, MNNG**			

**Mechanism**	**Targeted molecule(s)**	**First author, year**	**(Refs.)**

Phosphorylation activation of the autophagy signaling pathway is inhibited, negatively regulating the epithelial-mesenchymal transition cascade and uncontrolled cell proliferation induced by prolonged exposure to MNNG	PI3K/AKT signaling pathway	Liang *et al*, 2022	(29)

**C, High-fat diet**			

**Mechanism**	**Targeted molecule(s)**	**First author, year**	**(Refs.)**

Ectopic fat accumulation occurs, leading to increased expression of lysosomal LAMP2A and mitochondrial COXIV in the gastric mucosa, which induces lipotoxi-city and dysfunction in organelle biosynthesis, conferring cancer stem cell-like properties to the gastric mucosa	PI3K and β-catenin signaling pathways	Arita *et al*, 2016	(30)

**D, Deoxycholic acid**			

**Mechanism**	**Targeted molecule(s)**	**First author, year**	**(Refs.)**

Autophagy signaling pathway is abnormally activated, promoting the expression of CDX2, MUC2, Ki-67 and Cyclin-D1 protein and inducing the formation of an intestinal metaplasia phenotype	EGFR/PI3K/AKT/mTOR pathway	Sun *et al*, 2023	(31)

*H. pylori, Helicobacter pylori;* Rad51, RAD51 recombinase; miR, microRNA; BECN1, Beclin-1; BNIP3L, BCL2/adenovirus E1B 19 kDa interacting protein 3 like; AGS, human gastric adenocarcinoma; MNNG, N-Methyl-N'-nitro-N-nitrosoguanidine; LAMP2A, Lysosomal-associated membrane protein 2A; COXIV, Cytochrome c oxidase subunit IV; CDX2, Cyclooxygenase-2; MUC2, Mucin 2, Oligomeric Mucus/Gel-Forming.

**Table II.** Therapeutic agents for autophagy-mediated intestinal metaplasia.

Agent	Intervention strategy	Mechanism of action	Study type	First author, year	(Refs.)
Metformin	Inhibition of autophagy	Suppresses the PI3K/AKT/mTOR/HIF-1α signaling pathway, slowing the process of intestinal metaplasia in inflammation and apoptosis	Animal study (Atp4a^−/−^ mouse model)	Hu *et al*, 2024	(95)
Xiaojianzhong decoction	Activation of autophagy	Ameliorates gastric mucosal hypoxia and modulates the PI3K/AKT/mTOR and p53/AMPK/ULK1 signaling pathways to suppress aberrant autophagy and glycolysis in gastric precancerous lesions, thereby achieving effective treatment	Animal study (MNNG-induced Wistar rat model)	Zhang *et al*, 2023	(96)
Panax Notoginseng saponins	Activation of autophagy	Inhibits the PI3K/AKT/mTOR signaling pathway to enhance autophagy in gastric mucosal cells, improving incomplete intestinal metaplasia and dysplastic lesions	Animal study (GPL-MIRI mouse model)	Wang *et al*, 2024	(97)
*Celastrus orbiculatus*	Inhibition of autophagy	Modulates the PDCD4/ATG5 signaling pathway to suppress autophagy in gastric epithelial cells, ameliorating gastric mucosal damage and cell dysplasia in rats	Animal (MNNG-induced SD rat model) and *in vitro* study (GES-1 cells)	Zhu *et al*, 2024	(98)
Astragaloside IV	Inhibition of autophagy	Regulates p53 expression to activate the Ambra1/Beclin1 complex in gastric precancerous lesions, thereby protecting against gastric mucosal injury	Animal study (MNNG-induced Wistar rat model)	Cai *et al*, 2018	(99)
Cinnamaldehyde	Inhibition of autophagy	Modulates the downstream target mTOR via the PI3K/AKT signaling pathway to inhibit autophagy in gastric epithelial cells, counteracting gastric mucosal damage	Animal study (aspirin-induced gastric mucosal injury in C57BL/6 mice) and *in vitro* study (GES-1 cells)	Yan *et al*, 2024	(100)
Eudesmin	Inhibition of autophagy	Decreases the expression of LC-3B, caspase-3, caspase-9, caspase-8, Bax and Bid proteins, inhibiting *Helicobacter pylori*-induced apoptosis and autophagy	Animal (*Helicobacter pylori*-infected C57BL/6 mouse model) and *in vitro* study (AGS cells)	Yang *et al*, 2018	(101)
Astaxanthin	Activation of autophagy	Activates AMPK and down-regulates its downstream target mTOR to induce autophagy, suppressing *Helicobacter pylori*-induced apoptosis	*In vitro* study (AGS cells)	Lee *et al*, 2020	(102)
Glycyrrhizin	Activation of autophagy	Restores autolysosomal function and enhances autolysosome formation, inhibiting *Helicobacter pylori* infection	*In vitro* study (AGS cells)	Khan *et al*, 2023	(103)
Vitamin D3	Activation of autophagy	Activates the PDIA3 receptor to restore lysosomal degradation function, promotes nuclear translocation of the PDIA3-STAT3 protein complex and upregulates the MCOLN3 channel, leading to enhanced lysosomal Ca^2+^ release, normalized lysosomal acidification and eradication of *Helicobacter pylori*	Clinical and *in vitro* study (HFE145 and GES-1 cells)	Hu *et al*, 2019	(104)

HIF, hypoxia-inducible factor; ULK1, Unc-51 Like Autophagy Activating Kinase 1; MNNG, N-Methyl-N'-nitro-N-nitrosoguanidine; GPL-MIRI, Gastric Precancerous Lesion-Myocardial Ischemia-Reperfusion Injury; Bid, BH3 interacting domain death agonist; PDIA3, Protein disulfide isomerase family A member 3; MCOLN3, Mucolipin TRP cation channel 3.

## Data Availability

Not applicable.
